# Increased Ca2 + transport across the mitochondria-associated membranes by Mfn2 inhibiting endoplasmic reticulum stress in ischemia/reperfusion kidney injury

**DOI:** 10.1038/s41598-023-44538-0

**Published:** 2023-10-12

**Authors:** Shun Wang, Xiaohong Sang, Suhua Li, Wenjun Yang, Shihan Wang, Haixia Chen, Chen Lu

**Affiliations:** Nephrology Center, The First Affiliated Hospital of Xinjiang Medical University, Xinshi District, Urumqi, 830054 China

**Keywords:** Diseases, Medical research

## Abstract

Renal ischemia/reperfusion (I/R) injury, which leads to acute kidney injury (AKI), is a major cause of morbidity and mortality in a variety of clinical situations. This study aimed to investigate the protective role of Mfn2 during renal I/R injury. Overexpression of Mfn2 in NRK-52E rat renal tubular epithelial cells and rats, then we constructed hypoxia reoxygenation (H/R) cells and I/R rat model. Apoptosis, ROS, ATP, Ca^2+^ levels in cells and rats, as well as renal tissue and functional injury in rats were detected respectively. Endoplasmic reticulum (ER) stress was further examined in cells and rats. The morphological changes of mitochondria-associated ER membranes (MAMs) were also detected. Mfn2 expression is reduced in H/R-treated NRK-52E cells and renal tissue of I/R rats. At the cellular level, overexpression of Mfn2 promoted cell proliferation, inhibited cell apoptosis, attenuated mitochondrial damage and Ca^2+^ overload, and ER stress. In addition, Mfn2 also restored the MAMs structure. In vivo experiments found that overexpression of Mfn2 could improve renal function and alleviate tissue injury. Concomitant with elevated Mfn2 expression in the kidney, reduced renal cell apoptosis, restored mitochondrial function, and reduced calcium overload. Finally, ER stress in rat kidney tissue was alleviated after overexpression of Mfn2. These results reveal that Mfn2 contributes to ER stress, mitochondrial function, and cell death in I/R injury, which provides a novel therapeutic target for AKI.

## Introduction

Acute renal injury (AKI) has become a global public health problem and is associated with a high mortality rate^1^. AKI is characterized by a rapid decline in renal function over a short period of time, an increase in serum creatinine levels, and a decrease in urine production^2^. Over the past few decades, the incidence of AKI has gradually increased, and unfortunately, there are no effective interventions to improve the prognosis of established AKI, which requires a better understanding of its underlying pathophysiology. Due to the lack of effective treatments, AKI is often associated with renal dysfunction and ultimately leads to progressive nephron loss and end-stage renal disease^3^. Renal ischemia/reperfusion (I/R) injury is a common and critical factor for AKI in a variety of clinical situations and is a major cause of kidney transplant dysfunction^4^. Although the incidence of AKI caused by I/R injury is increasing year by year, strategies to effectively prevent or treat AKI are still limited.

I/R injury is a very complex process that usually includes two distinct pathophysiological phases: ischemia and reoxygenation^5^. AKI caused by I/R injury has a complex pathogenesis involving immune inflammatory response, oxidative stress, endoplasmic reticulum (ER) stress as well as apoptosis^6,7^. The kidney is particularly vulnerable to ischemia/hypoxia, and extensive molecular degeneration by oxidative stress has been implicated in the progression of acute renal cell injury^8^. Ischemia leads to dysfunction of the mitochondrial electron transport chain, whereas reactive oxygen species (ROS), nitrogen species production, and inflammatory response are amplified when blood supply is restored.

Stimulation of the ER by I/R and ROS can lead to the production of large amounts of unfolded or misfolded proteins and initiation of the unfolded protein response (UPR), ultimately resulting in ER stress^9^. Numerous studies have shown that renal I/R injury can induce ER stress and lead to AKI^10,11^. In addition, mitochondria and ER have a very close relationship in structure and function. The most important coupled organelles of the ER are mitochondria, and their binding sites are known as mitochondria-associated ER membranes (MAMs)^12^. Mitochondria and endoplasmic reticulum are physiologically interconnected through MAMs and participate in a variety of cellular biological and pathological processes^13^. Required to reduce endoplasmic reticulum stress and mitochondria mediated apoptosis in renal I/R injury, thereby reducing inflammatory responses and AKI. Mitofusin 2 (Mfn2) is located on MAMs and regulates ER and mitochondrial functions such as mitochondrial fusion and fission, calcium balance, and inflammatory responses^14^. However, whether Mfn2 participates in the apoptotic role in AKI by affecting MAMs dynamics is not clear.

Therefore, in this study, Mfn2 protein was overexpressed on cells and animal models to explore its effect on dynamic changes of mitochondria, ER stress and MAMs.

## Materials and methods

### Cell culture and Mfn2 overexpression

NRK-52E rat renal tubular epithelial cells (Yaji biological, Shanghai, China) were cultured in DMEM (Gibco, GI, USA) + 10% fetal bovine serum (FBS) + 1% penicillin streptomycin at 37 °C in 5% CO_2_.

Adv-Mfn2 or negative control (NC) virus GV314 (Genechem, Shanghai, China) were transfected into NRK-52E cells with MOI = 100 using viral coinfection reagents (Genechem) for 72 h. Cells were then harvested for quantitative reverse transcription-polymerase chain reaction (qRT-PCR) and Western blot to detect expression of Mfn2.

### qRT-PCR

Total RNA was extracted using Trizol Reagent (Ambion, MA, USA) from NRK-52E cells. RNA was then reverse transcribed into complementary DNA (cDNA) using the 5X All-In-One RT MasterMix (Ambion). The qRT-PCR was performed using cDNA as template with EvaGreen Express 2 × qPCR MasterMix-Low Rox (Ambion). GAPDH used as endogenous control to calculate expression of Mfn2 through 2^−ΔΔCt^ method. The primer sequences used in this study as follows: Mfn2 forward GAAGAAGAGTGTCAAGACCGTG, reverse CAGGCAAAACTTATCAATCCAG; GAPDH forward CAGGGCTGCCTTCTCTTGTG, reverse GATGGTGATGGGTTTCCCGT.

### Hypoxia reoxygenation (H/R) model establishment

Transfected or untransfected NRK-52E cells were incubated in a 37 °C 5% CO_2_ for 24 h, the DMEM medium (Gibco) without FBS or glucose was replaced and cultured in a saturated three gas incubator with 95% N_2_ + 5% O_2_ at 37 °C. After 6 h of hypoxic incubation, DMEM medium containing FBS and glucose was added and cultured in the 5% CO_2_ to continue the incubation for 6 h, 12 h, and 24 h of reoxygenation. The control group was incubated normally for the corresponding time in a 5% CO_2_ incubator.

### Cell proliferation assay

We used cell counting kit-8 (CCK8; TransGen Biotech, Beijing, China) to detect cell proliferation. A total of 5 × 10^4^ cells/mL of NRK-52E cells were seeded into 96 well plates (100 μL/well) and cultured overnight for H/R treatment after cell attachment. The medium in the wells was discarded, and 100 μL of 10% CCK8 solution was added to each well, then continue to culture in the 5% CO_2_ incubator. The OD value at 450 nm was measured with a microplate reader (Bio-Rad) after cultured for 1 h.

### Transmission electron microscopy

After cultured for 48 h, cells were fixed with 2% glutaraldehyde/0.1 M phosphate buffer (pH 7.4). Fixed cells were embedded in epoxy resin and cut into 60–80 nm ultrathin sections. Ultrathin sections were taken with a transmission electron microscope (TEM) after staining with uranyl acetate and lead citrate. Image-pro plus 6.0 (Media Cybernetics, MD, USA) was used to measure the distance of MAM, and to count the number of cristae.

### Construction of renal ischemia–reperfusion injury model

Sprague–Dawley male rats aged 6–8 weeks (weight 180–260 g) were provided by the experimental animal center of Xinjiang Medical University. The rats were housed at room temperature of 22 ± 2 °C, and with free access to food and water. Animal experimental were complied with the ARRIVE guidelines and were in compliance with the National Institutes of Health Guide for the Care and Use of Laboratory Animals. All rats were randomly divided into four groups: sham group (n = 24), renal ischemia–reperfusion (I/R; n = 27), renal I/R + adv-Mfn2 (n = 27), and renal I/R + adv-NC (n = 27). This research was approved by the Animal Care and Use Committee in the First Affiliated Hospital of Xinjiang Medical University (20190225-48).

Rats were anesthetized with by 4% phenobarbitone intraperitoneal injection. Only the right kidney was excised from rats in the sham group. Rats in the renal I/R group were excised the right kidney and released hemostats after clamping the left renal pedicle for 45 min. Tail vein injections 5 × 10^9^ PFU /rat of adv-Mfn2 or adv-NC, and 7 days later, the right kidney was excised and the left renal pedicle was clipped for 45 min before releasing hemostats. The rats in each group were sacrificed under anesthesia at 12 h, 24 h, and 72 h after surgery, respectively. Blood and left kidney tissue samples were collected.

### Detection of biochemical indexes

Whole blood was separated into serum and the contents of creatinine (Cr), blood urea nitrogen (BUN) and neutrophil gelatinase associated lipocalin (NGAL) in serum samples were detected by Enzyme Linked Immunosorbent Assay (ELISA) Kits (Nanjing jiancheng) according to manufacturer’s instructions.

### Histopathological observations

Kidney tissues were paraffin embedded after fixation in 10% formalin, then renal tissue sections (4 μm) were prepared. To assess the renal histoarchitecture and level of inflammation, sections were stained with hematoxylin and eosin (HE). Images were taken using a light microscope (Nikon) for observation. The degree of pulmonary inflammation was evaluated using the following levels in HE stained sections: 0 = no inflammatory response, 1 = mild inflammation, 2 = moderate inflammation, and 3 = severe inflammation.

Sections were stained with Masson's stain to assess renal fibrosis. Images were taken using a light microscope (Nikon) for observation. The proportion of renal tubulointerstitial collagen fibers relative to the total section area and classified as follows: 0 (nil), 1 (< 25%), 2 (25–50%), 3 (50–75%), and 4 (> 75% of tubulointerstitial fields).

### Immunohistochemical staining

Slices were subjected to antigen repair using high temperature and pressure. We used 5% bovine serum albumin to block non-specific binding sites on sections to reduce non-specific antibody binding. Specific antibody against proliferating cell nuclear antigen (PCNA; Santa Cruz, CA, USA) was added to the slices and incubated at 4 °C for 12 h. The unbound primary antibody was washed out and then added the HRP labeled secondary antibody. Subsequently, diaminobenzidine was added to the slices, and the substrate precipitated under the action of enzymes, revealing the location of the antigen. DAPI is used for nuclear staining. The results were quantified by counting the number of PCNA positive cells to the total number of cells from 10 randomly selected fields.

### Apoptosis

NRK-52E cells were digested with pancreatin and prepared as single-cell suspensions of 5 × 10^4^ cells/mL and seeded into 6-well plates (2 mL/ well). After H/R treatment, cells were collected and 5 μL Annexin V-PE (BD, WA, USA) and 10 μL 7-AAD (BD) were added and mixed gently. Incubate for 15 min at 4 °C in the dark. Flow cytometry was performed within 30 min to detect cell apoptosis.

Kidney tissues were paraffin embedded after fixation in 10% formalin. Renal tissue sections (5 μm) were prepared for performing terminal deoxynucleotidyl transferase dUTP nick-end labeling (TUNEL; Boster Biological Technology, Wuhan, China) staining to assess apoptosis. The results were expressed as the percentage of TUNEL positive cells to the total number of cells from 10 randomly selected fields.

### Mitochondrial membrane potential, and reactive oxygen species assay

NRK-52E cells were digested with pancreatin and prepared as single-cell suspensions of 5 × 10^4^ cells / mL and seeded into 6-well plates (2 mL/ well). For mitochondrial membrane potential detection, after H/R treatment, cells were collected and resuspended by adding 500 μL JC-1 staining solution (Nanjing jiancheng, Nanjing, China) and incubated at 37 °C for 30 min. Flow cytometry was performed within 30 min.

Fresh kidney tissues were ground, and pre-cooled PBS washed 2 times. Single-cell suspension of 1 × 10^5^ cells/mL was prepared. Then 1 × 10^5^ H/R NRK-52E cells and kidney single-cell suspension were added 2 μL DCFH-DA at a final concentration of 10 μM DCFH-DA. Cells were collected after incubation at 37 °C for 30 min, and resuspended in PBS. Fluorescence intensity detection was performed with excitation wavelength of 500 nm and emission wavelength of 525 nm using flow cytometry to detect reactive oxygen species (ROS).

### LDH and ATP assays

Levels of lactate dehydrogenase (LDH) in the cell supernatants was measured using LDH assay kit (Nanjing jiancheng) according to the manufacturer's guideline. The OD value at 450 nm was measured with a microplate reader (Bio-Rad).

Boiling double distilled water was added to the kidney tissue to make a 10% homogenate, which was then placed in a water bath and boiled for 10 min. Centrifuge at 1200 g for 10 min and take the supernatant. Adenosine triphosphate (ATP) levels in H / R NRK-52E cells and supernatant were measured using ATP assay kit (Nanjing jiancheng) according to the manufacturer's guideline. The OD value at 636 nm was measured with a microplate reader (Bio-Rad).

### Measurement of Ca^2+^

Cells at a final concentration of 1 × 10^5^ cells/mL were seeded into 6-well plates and subjected to H/R intervention. Fresh kidney tissues were ground, and pre-cooled PBS washed 2 times. Single-cell suspension of 1 × 10^5^ cells/mL was prepared. For detection of intracellular Ca^2+^ concentration, 4 μL Fluo-3 AM (Solarbio, Beijing, China) was added to H/R NRK-52E cells and kidney single-cell suspension, and the Fluo-3 AM final concentration was 5 μM. For detection of mitochondrial Ca^2+^ concentration, 4 μL Rhod-2 AM (Thermo Scientific, MA, USA) was added to H/R NRK-52E cells and kidney single-cell suspension, and the Rhod-2 AM final concentration was 5 μM. For detection Ca^2+^ concentration in ER, 4 μL Mag-Fluo-4 AM (Thermo Scientific) was added to H/R NRK-52E cells, and the Mag-Fluo-4 AM final concentration was 5 μM. Cells were incubated at 37 °C for 30 min and washed twice with PBS. The incubation was continued by adding 2 mL PBS into the incubator at 37 °C for 30 min. Samples were collected, and 500 μL PBS was added to resuspend the cells for fluorescence intensity detection at excitation wavelength of 500 nm and emission wavelength of 525 nm using flow cytometry.

### Western blot

Kidney tissues at 24 h after I/R were ground into homogenates in liquid nitrogen. Protein was extracted from tissues and cells using Radio Immunoprecipitation Assay (RIPA) buffer with protease inhibitors on ice. The concentration of protein was detected using Easy II Protein Quantitative Kit (TransGen Biotech). Proteins (50 μg) were separated on 12% polyacrylamide gels using electrophoresis. Then proteins were transferred to PVDF membranes. After blocking with nonfat milk, the membranes were incubated with primary antibodies (Mfn2, GRP78, PERK, p-PERK, GRP75, MICU1, IP3R1, MCU, VDAC1, and β-actin; Cell Signaling Technology, MA, USA) overnight at 4 °C. Subsequently, the membranes were incubated with HRP-conjugated IgG antibody at room temperature for 2 h. The bands were observed with ECL solution on chemiluminescence system (Bio-Rad). Protein relative expression was calculated by ImageJ software.

### Statistical analyses

All data were expressed as mean ± standard deviation. GraphPad Prism software was used to analyze the data of each group. Statistical analysis was performed by one-way ANOVA between groups. *P* < 0.05 indicated significant difference.

### Ethics approval and consent to participate

All experimental protocols were approved by the Animal Care and Use Committee in the First Affiliated Hospital of Xinjiang Medical University (20190225-48).

## Results

### Mfn2 overexpression changes apoptosis and mitochondrial function

**Figure 1:**
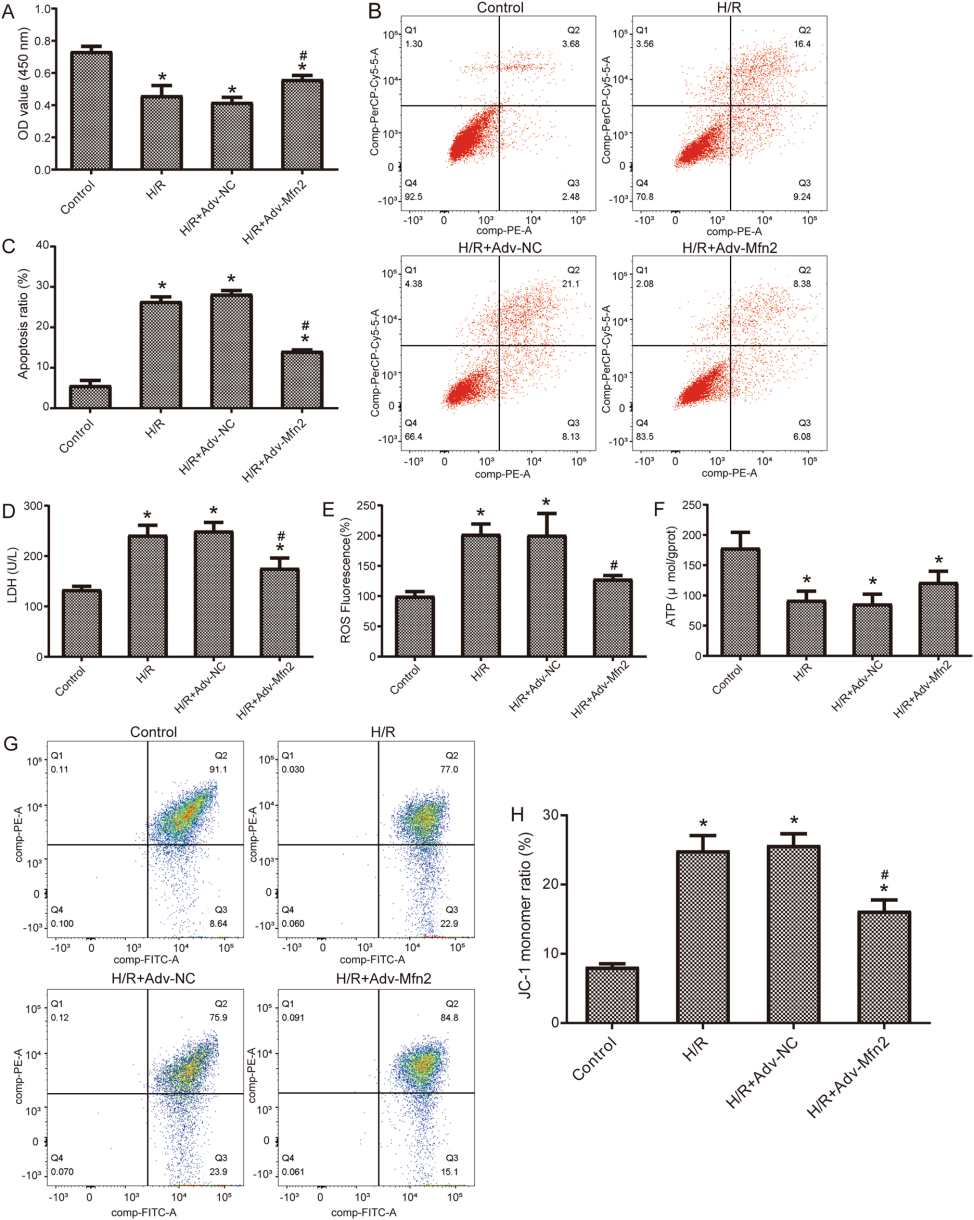
Overexpression of Mfn2 altered NRK-52E cell injury and mitochondrial function. (**A**) Detection of cell proliferation by CCK8 in H/R exposure and overexpression of Mfn2. (**B**) Apoptosis was detected by flow cytometry in H/R exposure and overexpression of Mfn2. (**C**) Statistical results of cell apoptosis ratio. (**D**) The level of LDH in H/R exposure and overexpression of Mfn2. (**E**) Intracellular ROS levels measured by flow cytometry in H/R exposure and overexpression of Mfn2. (**F**) The level of ATP in H/R exposure and overexpression of Mfn2. (**G**) Mitochondrial membrane potential was detected by flow cytometry in H/R exposure and overexpression of Mfn2. (**H**) Statistical results of JC-1 monomer ratio. *P < 0.05 compared to Control; #* P* < 0.05 compared to H/R + Adv-NC. H/R, hypoxia reoxygenation; LDH, lactate dehydrogenase; ROS, reactive oxygen species; ATP; adenosine triphosphate.

We first identified the expression of Mfn2 in NRK-52E cells for different reoxygenation times after hypoxia by Western blot. The lowest Mfn2 expression level was observed at 6 h in reoxygenation after hypoxia, which was significantly lower than that of the control group (Figure S1A). We then overexpressed Mfn2 (Figure S1B, C). To identify the influence of Mfn2 in H/R injury, we examined cell proliferation (Fig. 1A). Compared with control group, cell proliferation in H/R treated cells were decreased. The proliferation of cells treated with H/R was significantly higher after overexpression of Mfn2 compared with transfection of negative control (NC) virus. In the result of apoptosis assays, we found that apoptosis was significantly elevated by H/R, whereas it was reduced after overexpression of Mfn2 (Fig. 1B,C). LDH is significantly increased in H/R treated cells, then it decreases after overexpression of Mfn2 (Fig. 1D). In addition, we also examined mitochondria related functions. The H/R treatment significantly promoted the level of ROS, which was decreased after overexpression of Mfn2 (Fig. 1E). The levels of ATP were significantly lower after H/R group than in control cells, and were upregulated after overexpression of Mfn2 (Fig. 1F). Mitochondrial membrane potential was also elevated after H/R treatment and decreased after overexpression of Mfn2 (Fig. 1G,H). The results suggested that the HR injury was partially alleviated after overexpressing Mfn2.

### Mfn2 attenuates ER stress in H/R exposure

**Figure 2:**
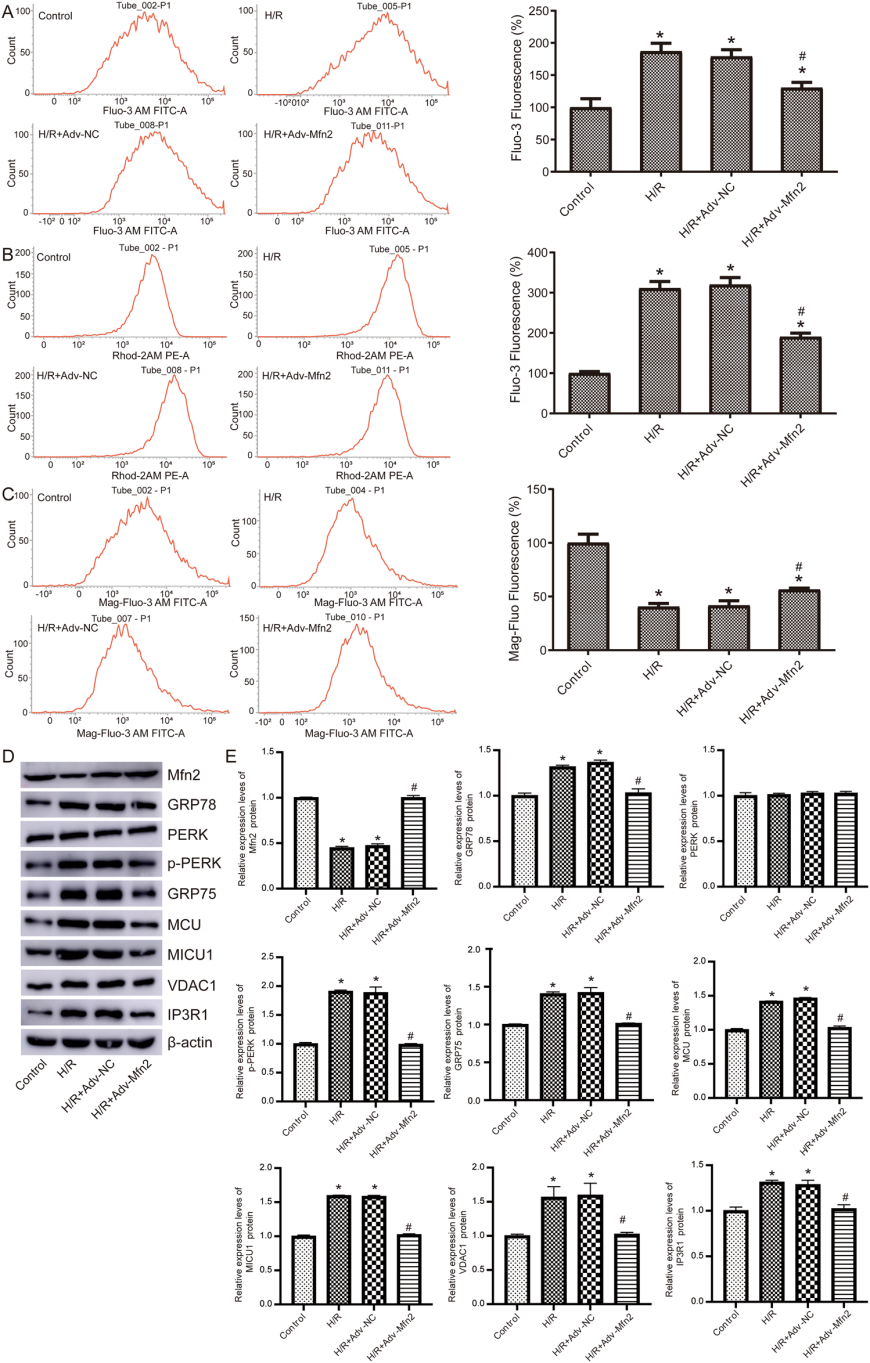
Mfn2 inhibits ER stress in H/R treated NRK-52E cells. (**A**) Intracellular Ca^2+^ concentration was detected by Fluo-3 AM flow cytometry. (**B**) Mitochondrial Ca^2+^ concentration was detected by Rhod-2 AM flow cytometry. (**C**) Ca^2+^ concentration in ER was detected by Mag-Fluo-4 AM flow cytometry. (**D**) The expression of ER stress related protein detected by western blot. Original blots are presented in Supplementary Fig. 2. (**E**) Statistical results of relative protein expression levels. *P < 0.05 compared to Control; #* P* < 0.05 compared to H/R + Adv-NC. H/R, hypoxia reoxygenation.

Further, we detected the level of Ca^2+^ in H/R treated NRK-52E cells. We detected the intracellular Ca^2+^ concentration and found that, compared with the control group, the Ca^2+^ concentration was significantly higher in the H/R group, and this was significantly decreased after overexpression of Mfn2 compared to H/R + Adv-NC group (Fig. 2A). Mitochondrial Ca^2+^ concentration was higher in H/R group than control, and lower in Mfn2 overexpressed H/R cells than H/R + Adv-NC group (Fig. 2B). Contrarily, Ca^2+^ concentration in ER decreased in the H/R cells and increased when overexpressing Mfn2 (Fig. 2C).

To determine the role of ER stress in NRK-52E H/R injury, we examined the expression of related proteins (Fig. 2D,E). After H/R, Mfn2 expression was reduced and the expression of GRP78, p-PERK, GRP75, MCU, MICU1, VDAC1, and IP3R1 was increased. When Mfn2 was overexpressed in HR cells, the expression of GRP78, p-PERK, GRP75, MCU, MICU1, VDAC1, and IP3R1 was significantly decreased. This suggests that Mfn2 may activate PERK to participate in ER stress and promote Ca^2+^ transport.

**Figure 3:**
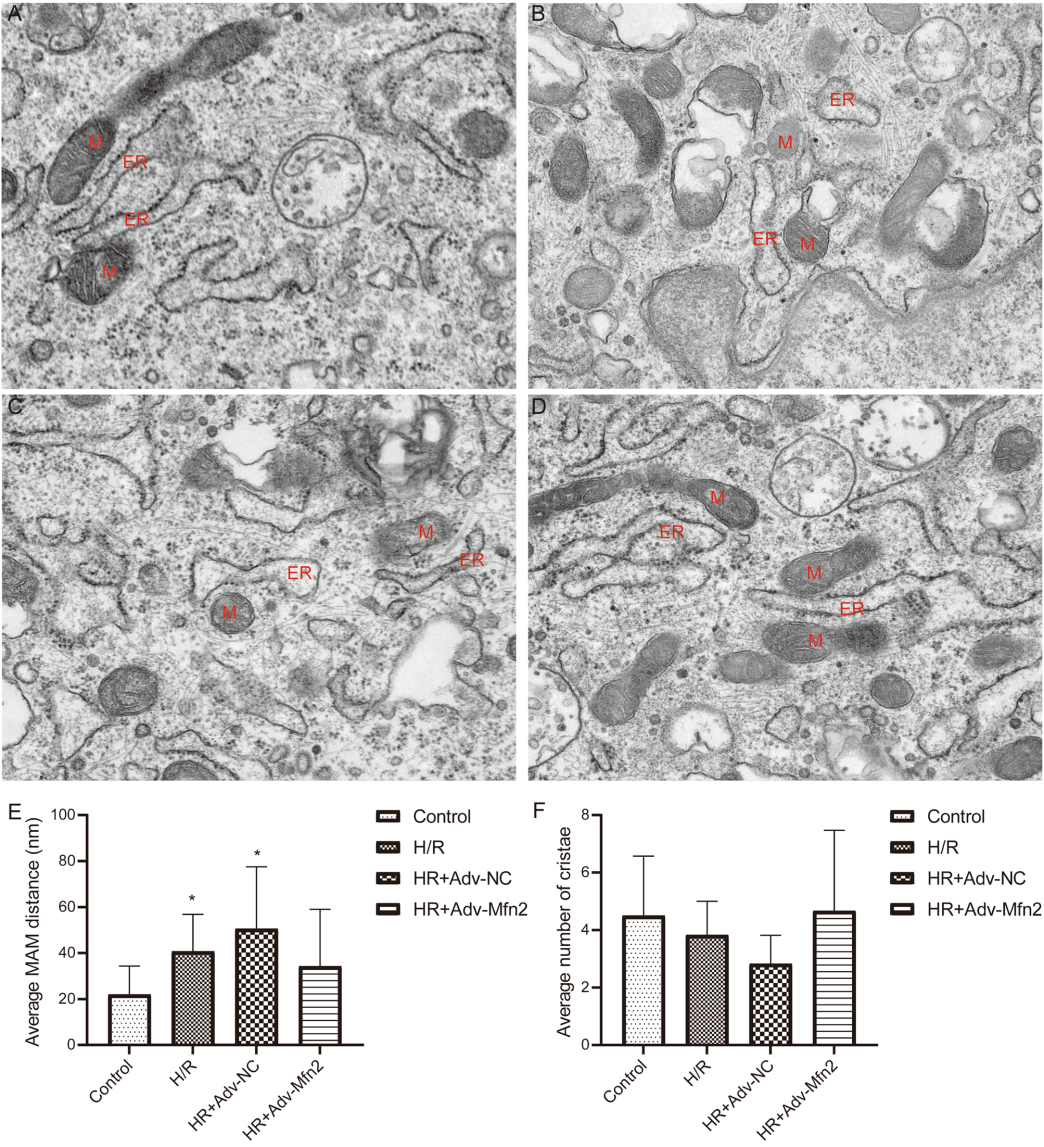
Transmission electron microscope analysis of endoplasmic reticulum and mitochondria in NRK-52E cells. Representative transmission electron microscope images of mitochondria-endoplasmic reticulum contacts in NRK-52E cells (**A**), H/R treated NRK-52E cells (**B**), H/R treated NRK-52E cells with Adv-NC (**C**), and H/R treated NRK-52E cells with Mfn2 overexpression (**D**). M, mitochondria; ER, endoplasmic reticulum. Bar = 500 nm. *P < 0.05 compared to Control. (**E**) The average distance of MAMs between the ER and mitochondria. (**F**) The average number of mitochondrial cristae.

The morphology and distance changes of mitochondria and ER in NRK-52E cells were observed by TEM. Mitochondria in normal controls were intact with clearly visible cristae, and the mitochondrial surface formed close contacts with the ER (Fig. 3A). The H/R injury group showed significant mitochondrial defects, with loose contacts formed on the mitochondrial surface (Fig. 3B,C). The distance of loose contact between ER and mitochondria was significantly shorter after overexpression of Mfn2 (Fig. 3D). Compared with the control group, the average distance between ER and mitochondria in H/R injury group was increased, while the number of cristae was decreased, then overexpression of Mfn2 improved these changes (Fig. 3E,F).

### Mfn2 overexpression attenuates renal injury after renal ischemia–reperfusion

**Figure 4:**
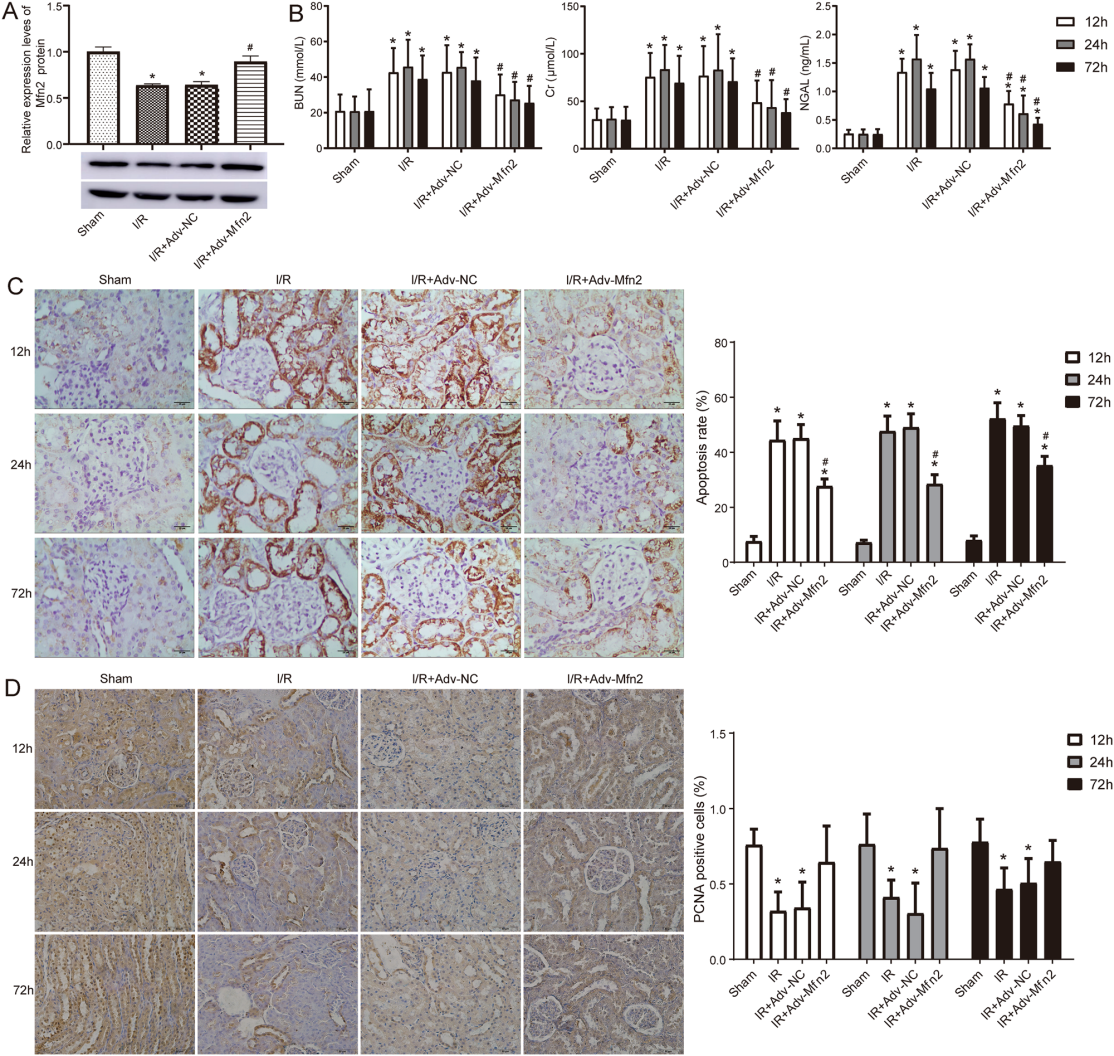
Mfn2 inhibits renal injury and apoptosis after ischemia–reperfusion. (**A**) The protein levels of Mfn2 detected by Western blot in sham and renal I/R model rat with adv-Mfn2 or adv-NC. Original blots are presented in Supplementary Fig. 3. (**B**) Serum BUN, Cr, and NGAL level in sham and renal I/R model rat with adv-Mfn2 or adv-NC at 12 h, 24 h, and 72 h after surgery. (**C**) Apoptosis was detected by TUNEL staining at 12 h, 24 h, and 72 h after surgery. The percentage of TUNEL positive cells represented the apoptosis rate. Bar = 25 μm. (**D**) Cell proliferation was detected by PCNA staining at 12 h, 24 h, and 72 h after surgery. The percentage of PCNA positive cells represented the proliferation rate. Bar = 50 μm. *P < 0.05 compared to Control; #* P* < 0.05 compared to I/R + Adv-NC. BUN, blood urea nitrogen; Cr, creatinine; NGAL, neutrophil gelatinase associated lipocalin; I/R, ischemia/reperfusion; PCNA, proliferating cell nuclear antigen.

Based on the results of preliminary experiments, we constructed the rat renal I/R model. We observed that down regulated expression of Mfn2 was induced in the kidney after I/R, which was significantly higher when injected with adv-Mfn2 (Fig. 4A). BUN, CRE, and NGAL, the markers of renal injury, were significantly elevated in the renal I/R model but were significantly downregulated in the group overexpressing Mfn2 (Fig. 4B). To examine the renoprotective effects of Mfn2 on apoptosis, PCNA and TUNEL staining was applied. Compared with the sham group, renal apoptosis of rats in the I/R group was obvious, and overexpression of Mfn2 effectively reduced the level of apoptosis (Fig. 4C). Compared with the sham group, renal proliferation in the I/R group was reduced, and overexpression of Mfn2 effectively increased the level of cell proliferation (Fig. 4D).

**Figure 5:**
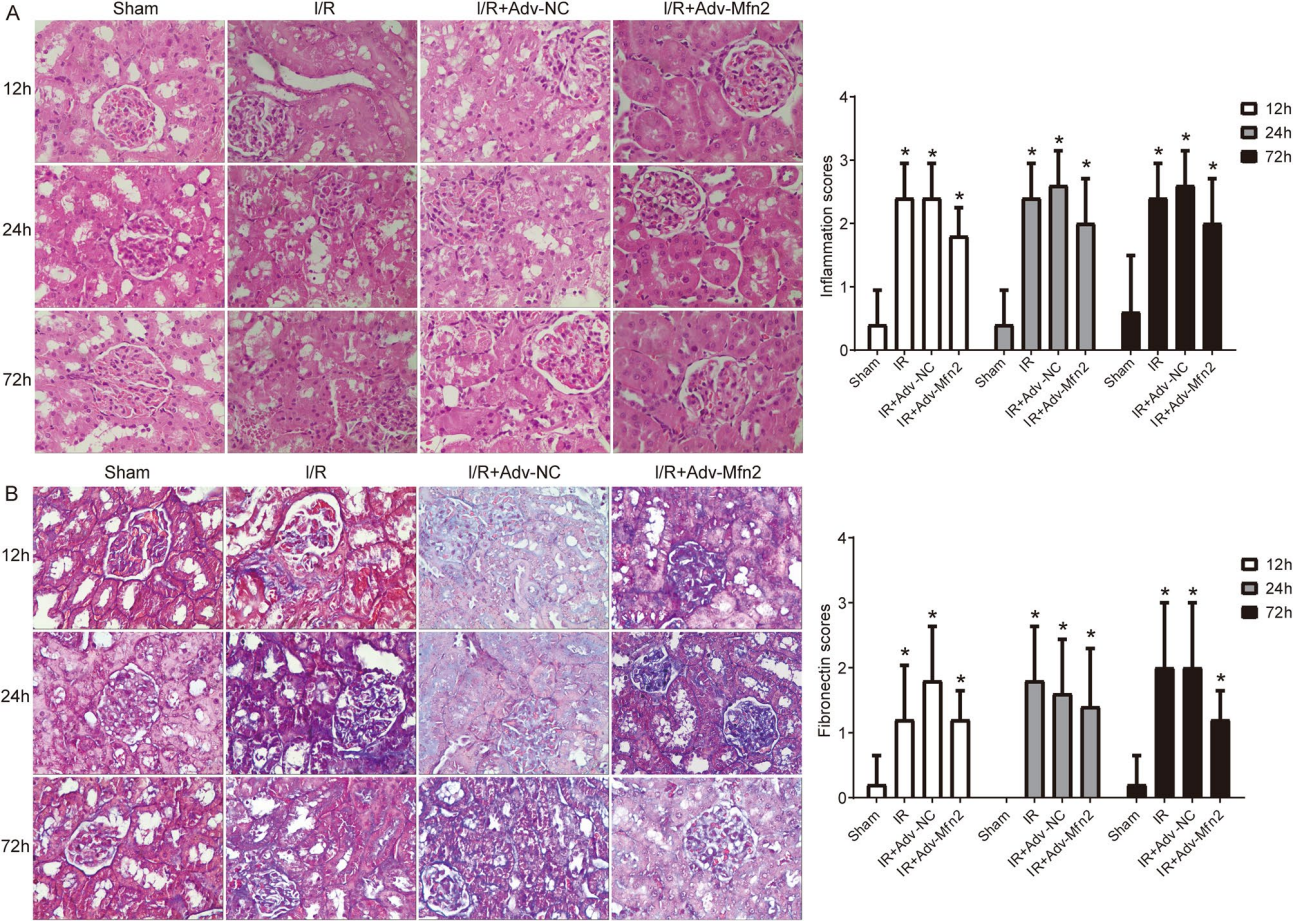
HE and Masson staining of kidney tissues. (**A**) Kidney stained with HE after renal I/R injury with adv-Mfn2 or adv-NC at 12 h, 24 h, and 72 h after surgery. (**B**) Kidney stained with Masson to detect chronic renal fibrosis after renal I/R injury with adv-Mfn2 or adv-NC at 12 h, 24 h, and 72 h after surgery. Bar = 25 μm. I/R, ischemia/reperfusion. *P < 0.05 compared to Control.

Next, HE staining confirmed that renal histomorphology, including disorganized architecture, focal hemorrhage, and scattered inflammatory cell infiltration exhibited in the I/R group were effectively improved by Mfn2 overexpression (Fig. 5A). Masson staining revealed that significant fibrosis was observed in the kidneys of I/R group, whereas the I/R + adv-Mfn2 group showed reduced fibrosis compared with the I/R + adv-NC group (Fig. 5B). These results confirmed that overexpression of Mfn2 effectively attenuated renal pathological damage in rat after I/R model establishment.

### Mfn2 inhibits ER stress and drives mitochondrial function

**Figure 6:**
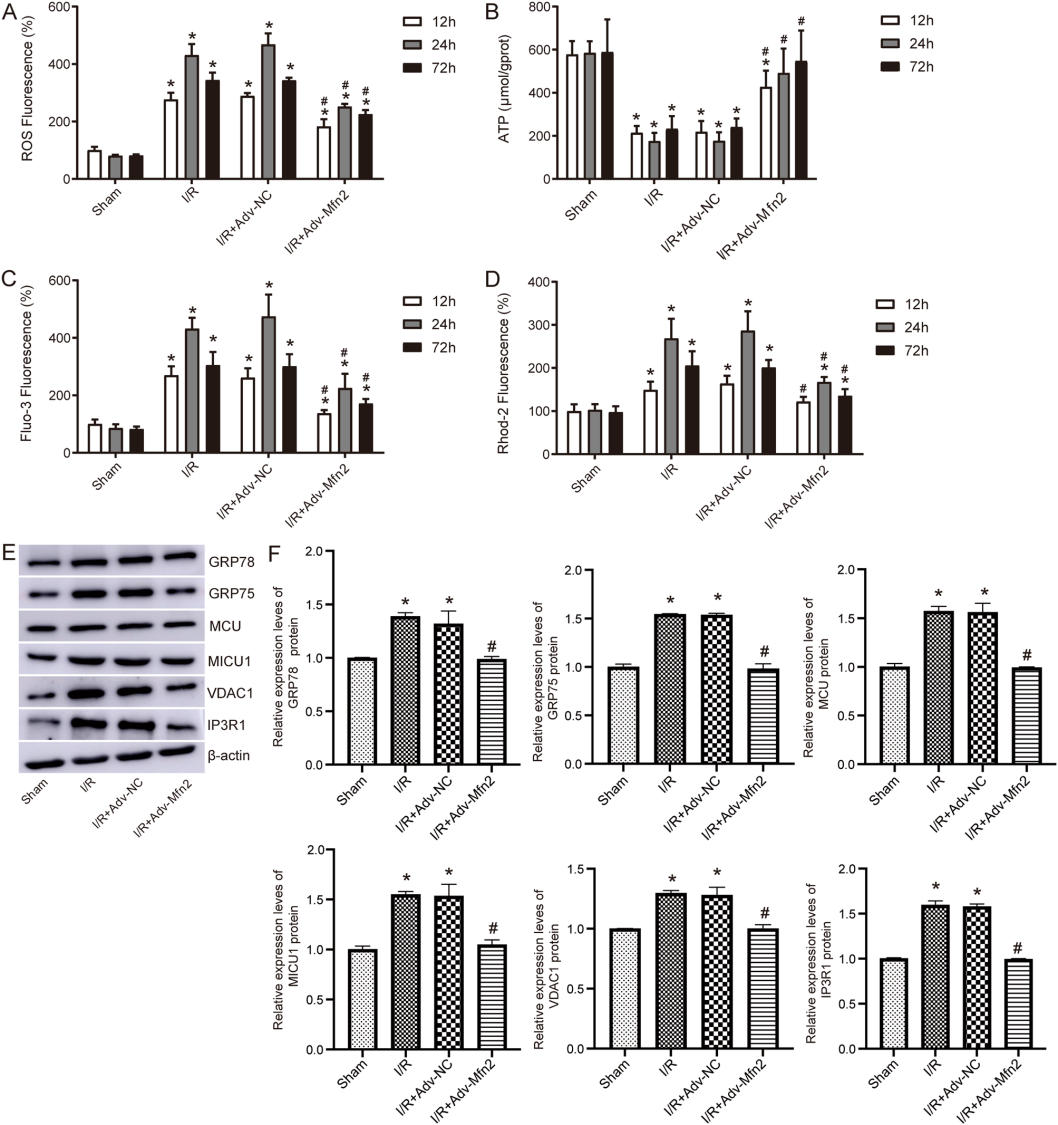
The effect of Mfn2 on Ca^2+^ transport and ER stress related proteins after I/R injury. (**A**) ROS levels in the kidney tissues measured by flow cytometry in renal I/R model rat with adv-Mfn2 or adv-NC. (**B**) The level of ATP in renal I/R model rat with adv-Mfn2 or adv-NC. (**C**) Intracellular Ca^2+^ concentration was detected by Fluo-3 AM flow cytometry. (**D**) Mitochondrial Ca^2+^ concentration was detected by Rhod-2 AM flow cytometry. (**E**) The expression of ER stress related protein detected by western blot at 24 h after renal I/R injury. Original blots are presented in Supplementary Fig. 4. (**F**) Statistical results of relative protein expression levels. *P < 0.05 compared to Control; #* P* < 0.05 compared to I/R + Adv-NC. ROS, reactive oxygen species; ATP; adenosine triphosphate; I/R, ischemia/reperfusion.

We aimed to reconfirm mitochondrial dysfunction in acute renal I/R injury. As shown in Fig. 6A, ROS levels in the kidney tissues of IR group were significantly higher than those in sham group, and which were reduced after overexpression of Mfn2. A significant decrease in ATP content was observed in the kidney tissue of the I/R group compared with the sham group, whereas an obvious increase in ATP content was observed in the I/R + adv-Mfn2 group (Fig. 6B). Examination of Ca^2+^ concentrations found that both intracellular Ca^2+^ concentrations (Fig. 6C) and intramitochondrial Ca^2+^ concentrations (Fig. 6D) in kidney tissue were significantly increased in I/R group compared with sham. Then, Ca^2+^ concentration was reduced after overexpression of Mfn2.

We determined the relationship between ER stress related proteins and Mfn2 in renal I/R injury by detecting protein expression (Fig. 6E,F). Expression of GRP78, GRP75, MCU, MICU1, VDAC1, and IP3R1 was increased in I/R group compared with sham, whereas the protein expression was significantly decreased after overexpression of Mfn2. These results indicated that Mfn2 protects against I/R renal injury by regulating Ca^2+^ transport and ER stress.

## Discussion

As a common and important cause of AKI, I/R injury has multiple mechanistic factors, and effective prevention and intervention methods are urgently needed. In the present results, we found that Mfn2 protein expression was elevated, activated endoplasmic reticulum stress, mitochondria dysfunction and Ca^2+^ overload occurred in H/R cells and I/R injury model. Cell apoptosis, kidney tissue morphology, endoplasmic reticulum stress, and mitochondrial function were obviously improved after Mfn2 overexpression. In addition, the protective effect of Mfn2 against renal I/R injury may be related to the preservation of MAM structure, which is an important finding of this study.

The results of the current study demonstrated that the levels of LDH and ROS were significantly increased and ATP levels were significantly decreased in cells subjected to H/R. In reperfusion injury, apoptosis is associated with increased LDH and ROS^15^. Excessive ROS produced by mitochondrial dysfunction is an important cause of oxidative damage caused by I/R^16^. Animal models of renal I/R injury are often used to mimic the pathological mechanisms and therapeutic means for AKI^17^. The present study demonstrated that I/R resulted in severe impairment of renal function, as shown by significantly increased bun, Cr, and NGAL levels in rats. Histological findings suggested that the I/R group showed severe histological changes, indicating renal damage. Previous studies have shown that I/R can lead to severe renal injury, which is often manifested clinically by increased bun and Cr levels and proteinuria^18^. NGAL expression is induced in renal tubular cells during kidney injury and represents a potential new biomarker for I/R-induced AKI^19^. The current study showed that Mfn2 overexpression reduced renal injury markers and ROS production, and alleviated renal tissue injury. In support of this, previous studies have shown that following I/R or H/R, Mfn2 levels are reduced and stabilization or elevation of Mfn2 levels can ameliorate reperfusion injury^20^.

The generation of ROS, which alters the cellular redox potential and thus leads to apoptosis, has been shown to occur following renal I/R^21^. Mfn2 deficiency in proximal tubular epithelial cells promotes apoptosis under stress conditions characterized by ATP depletion^22^. Our results highlight the role of Mfn2 in reducing renal cell apoptosis in I/R injury. Mfn2 is involved in the regulation of cell proliferation, apoptosis and many other different biological processes^23^. Up regulation of Mfn2 protein expression reduces apoptosis in hepatic I/R injury^24^.

In the current study, we found that Mfn2 overexpression significantly reduced Ca^2+^ overload in I/R injured renal cells and mitochondria. In addition to targeting mitochondria, Mfn2 also exists on the membrane of ER, which is considered to facilitate the transfer of Ca^2+^from the ER to adjacent mitochondria^25^. More importantly, Mfn2 plays an important role in maintaining or buffering cytosolic Ca^2+^ by taking up Ca^2+^ into mitochondria^26^. Therefore, Mfn2 mediated inter organelle communication of Ca^2+^ plays a crucial role in the pathophysiology of I/R.

Renal I/R injury induces abnormal mitochondrial morphology and increases the percentage of damaged mitochondria^27^. Mitochondrial fragmentation is a typical pathological feature of AKI, which implies imbalanced mitochondrial fission and fusion in kidney disease. The abnormal activation of Drp1 and the inhibition of Mfn2 are the main reasons for excessive Mitochondrial fission in the disease^28^. AKI, accompanied by excessive ROS production, often leads to the downregulation of mitochondrial fusion proteins Opa1 and Mfn2, as well as increase of mitochondrial fission proteins Drp1 and Fis1, which leads to the pathological increase of Mitochondrial fission, causing damage to renal tubular cells^29^. Mitochondria play a key role in apoptosis through their function in the production of ATP and reactive oxygen species and the balance of Ca^2+^^30^. When the expression of Mfn2 is downregulated in I/R injury, the fragmentation of mitochondria is increased in apoptotic cells^31^. In cells exposed to H/R, mitochondrial membrane potential and ATP content were partially restored by Mfn2 protein. In this study, we found by TEM that Mfn2 significantly reduced mitochondrial swelling and cristae fragmentation.

Mitofusins are downregulated during renal I/R injury, leading to disrupted mitochondrial dynamics^32^. Low Mfn2 protein expression in H9c2 cells after H/R treatment increased mitochondrial damage and oxidative stress^33^. Mfn2 is required for the normal recovery of mitochondria during reoxygenation phase and for the reconstruction of mitochondria network after injury^34^. In fact, Mfn2 maintains mitochondrial homeostasis in cardiomyocytes through fusion events and mitosis^35^. Reducing mitochondrial damage, inhibiting oxidative stress and apoptosis of renal tubular epithelial cells are important mechanisms in the treatment of AKI induced by I/R injury^36^.

In addition to the mitochondrial pathway, we found that endoplasmic reticulum stress occurred within the renal tissue subjected to I/R injury. Accumulating evidence suggests that ER stress and apoptosis are important steps in the pathogenesis of renal AKI^37^. Previous studies have shown that Mfn2 and mitochondrial dysfunction are upstream of ER stress, and a decrease in Mfn2 triggers ER stress^38^. Related studies have shown that elimination of Mfn2 can inhibit perk activation and lead to ER stress^39^. The PERK stabilizing binding protein IP3R reduces ER Ca^2+^ release and stabilizes Ca^2+^ concentration at mitochondrial ER contact sites, which in turn protects against ER stress^40^. Meanwhile, ER stress is a mediator of cytosolic calcium overload^41^. These results suggest that Mfn2 may activate PERK to participate in ER stress and promote Ca^2+^ transport.

Furthermore, TEM assay found that Mfn2 overexpression shortened the distance between mitochondria and endoplasmic reticulum. Mfn2, the linker connecting the endoplasmic reticulum and mitochondria, plays an important role in the formation of MAMs^42^. Several studies have shown that Mfn2 regulates mam dynamics and mitochondrial fusion under disease conditions^43^. Alterations in MAMs also increase levels of mitochondrial ROS^44^. Therefore, we speculate that the role of Mfn2 in reducing apoptosis in I/R injury may be mediated by inhibiting ER stress and promoting mam formation to protect mitochondria.

There are some limitations in the current study. First, although the I/R injury experiment lasted for 72 h, the effect of ER stress was only assessed at 24 h after AKI. Second, we did not detect ER stress and mitochondrial biogenesis dynamically, which may generate a vicious cycle. To fully understand the interplay of mitochondrial biogenesis and ER stress in the kidney after AKI. Furthermore, overexpression of Mfn2 ameliorated renal dysfunction after I/R injury, but whether side effects are produced is unknown.

## Conclusion

In conclusion, H/R-treated NRK-52E cells and renal I/R injury induce Mfn2 downregulation, leading to ER stress, mitochondrial dysfunction, MAM structure, and apoptosis. Our data highlight that overexpression of Mfn2 effectively ameliorates I/R renal injury. Therefore, elevating the expression of Mfn2 may play an important role in the rapid recovery of AKI induced by I/R injury.

### Supplementary Information

Supplementary Information.

## Data Availability

The data used to support the findings of this study are available from the corresponding author upon request.
