# Author Correction: Increased Ca2+ transport across the mitochondria-associated membranes by Mfn2 inhibiting endoplasmic reticulum stress in ischemia/reperfusion kidney injury

**DOI:** 10.1038/s41598-024-52771-4

**Published:** 2024-01-30

**Authors:** Shun Wang, Xiaohong Sang, Suhua Li, Wenjun Yang, Shihan Wang, Haixia Chen, Chen Lu

**Affiliations:** Nephrology Center, The First Afliated Hospital of Xinjiang Medical University, Xinshi District, Urumqi, 830054 China

Correction to: *Scientific Reports* 10.1038/s41598-023-44538-0, published online 12 October 2023

The original version of this Article contained an error in Figure 5 where the images of the I/R group for 72 hours were incorrectly placed on the I/R + adv Mfn2 group for 24 hours. The original Figure 5 and accompanying legend appear below.

**Figure 5:**
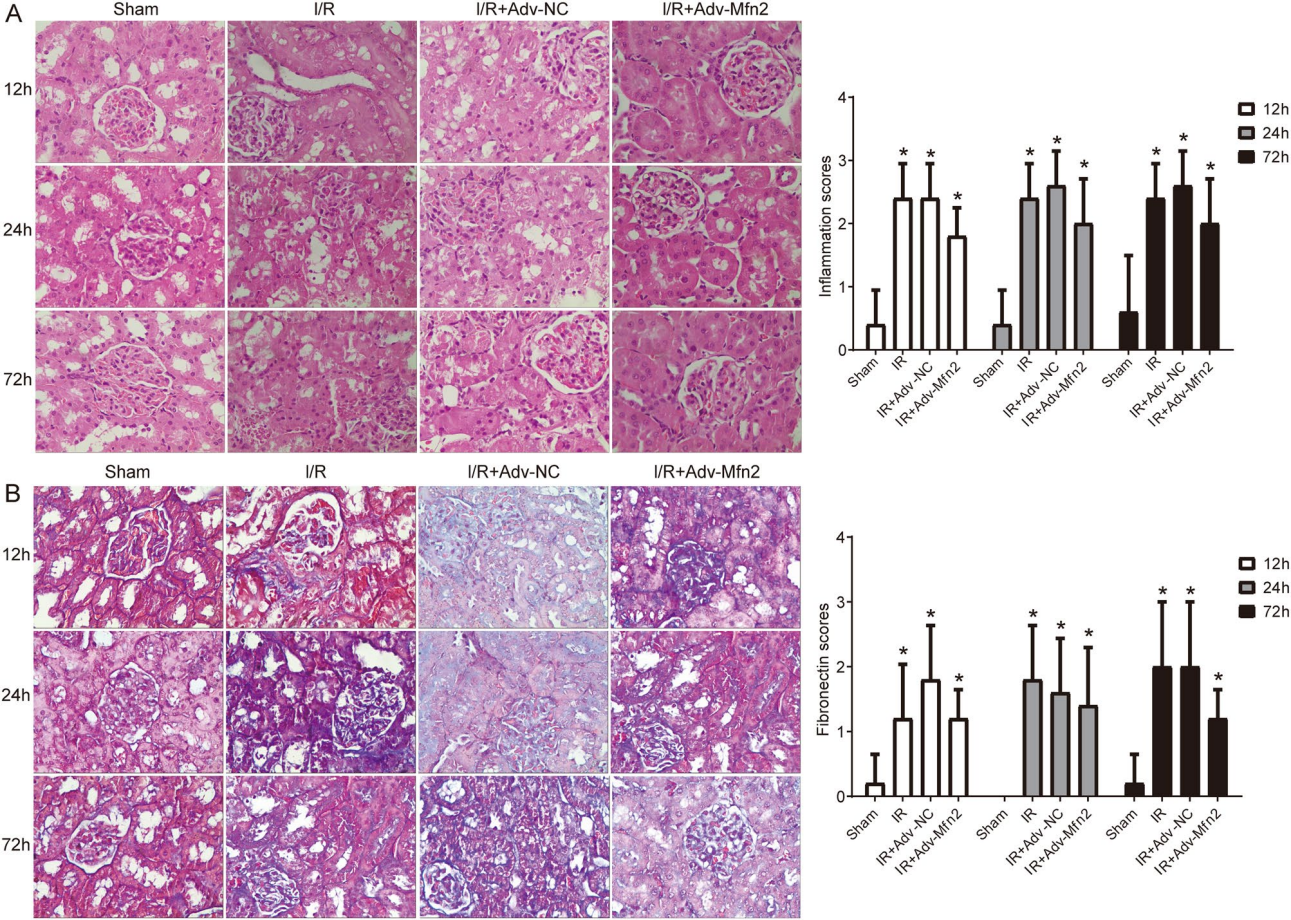
HE and Masson staining of kidney tissues. (**A**) Kidney stained with HE after renal I/R injury with adv-Mfn2 or adv-NC at 12 h, 24 h, and 72 h after surgery. (**B**) Kidney stained with Masson to detect chronic renal fibrosis after renal I/R injury with adv-Mfn2 or adv-NC at 12 h, 24 h, and 72 h after surgery. Bar = 25 μm. I/R, ischemia/reperfusion. *P < 0.05 compared to Control.

The original Article has been corrected.

