# Supplementary material for: Increased Ca2 + transport across the mitochondria-associated membranes by Mfn2 inhibiting endoplasmic reticulum stress in ischemia/reperfusion kidney injury
Source: Sci Rep. 2023 Oct 12;13:17257. doi: 10.1038/s41598-023-44538-0 (PMC10570331; doi:10.1038/s41598-023-44538-0)
Supplement: Supplementary file 1 — Supplementary Information. [file 41598_2023_44538_MOESM1_ESM.pdf]

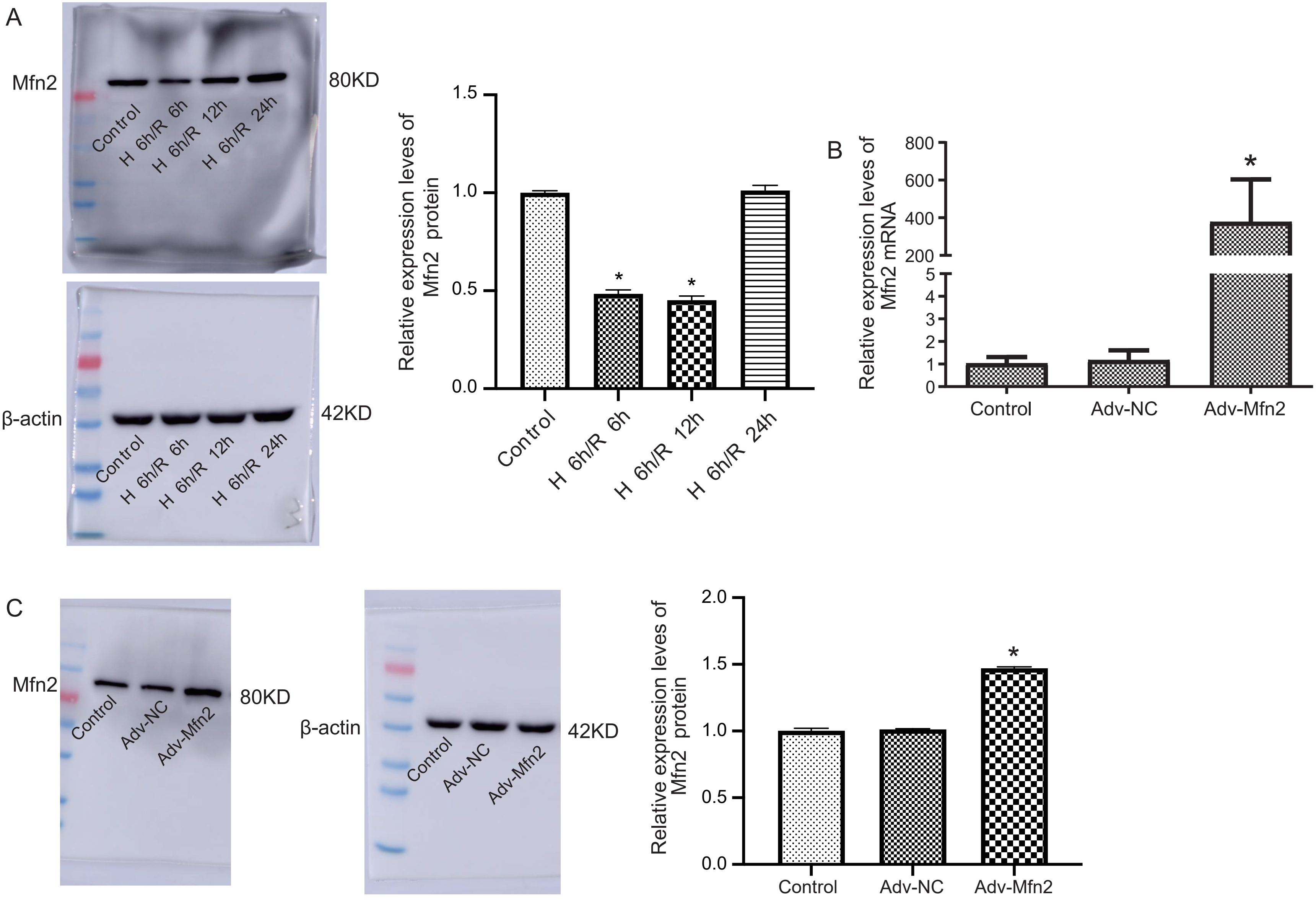

Figure S1. The expression of Mfn2 in NRK-52E cells. (A) The expression of Mfn2 in different reoxygenation times after hypoxic incubation. (B) The mRNA levels of Mfn2 after transfected with Adv-Mfn2 detected by qRT-PCR. (C) The protein levels of Mfn2 after transfected with Adv-Mfn2 detected by Western blot. *P < 0.05. H, hypoxia; R, reoxygenation.

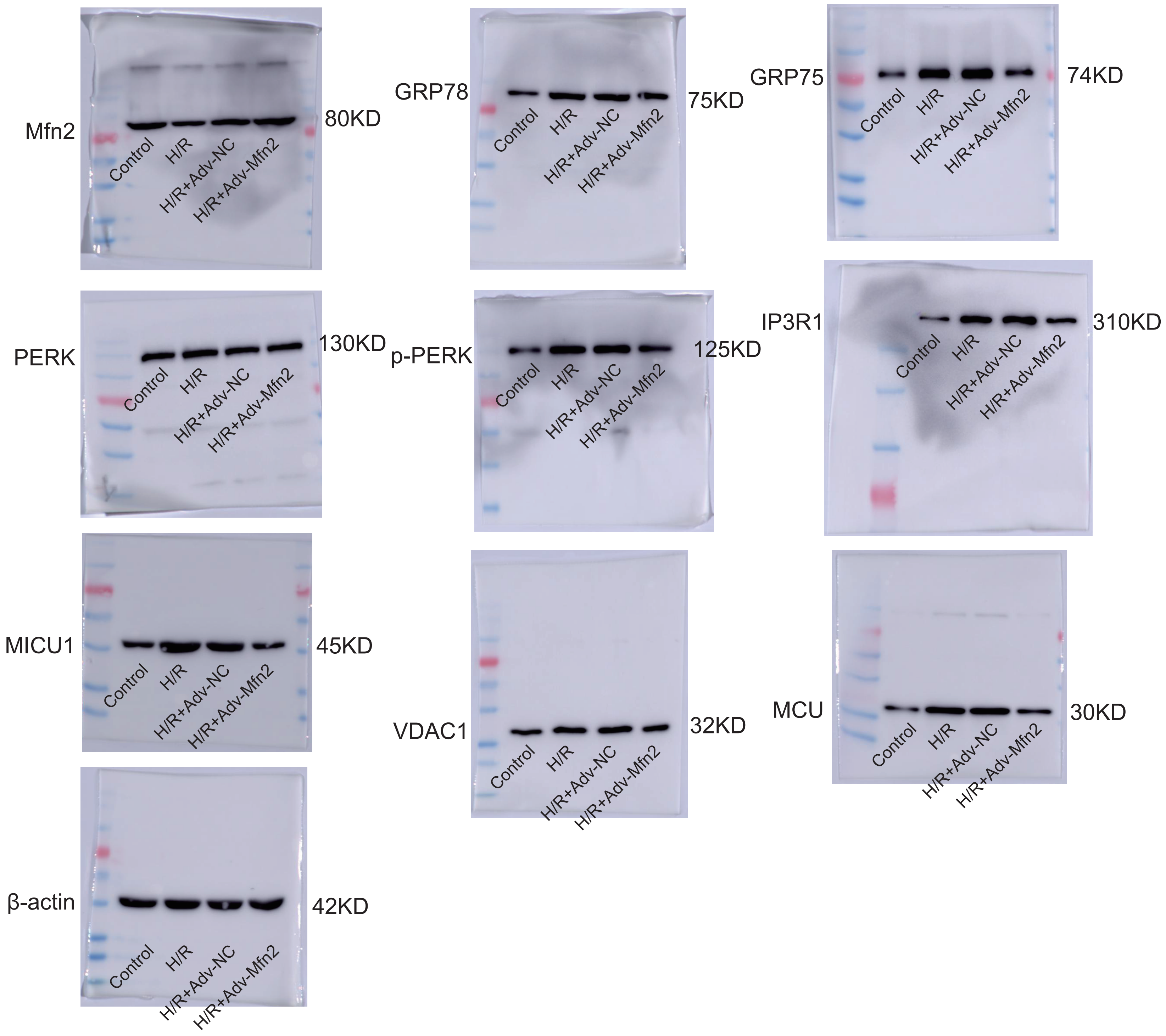

Figure S2. Images of expression of ER stress related protein detected by Western blot.

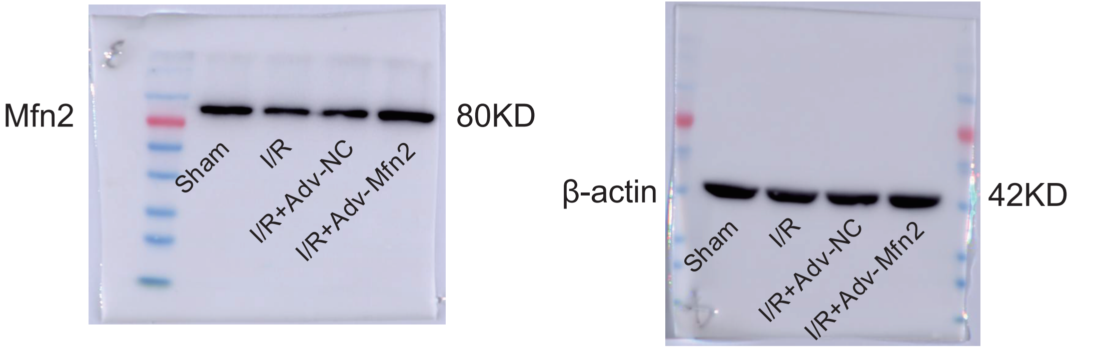

Figure S3. Images of the protein levels of Mfn2 detected by Western blot in sham and renal I/R model rat with adv-Mfn2 or adv-NC.

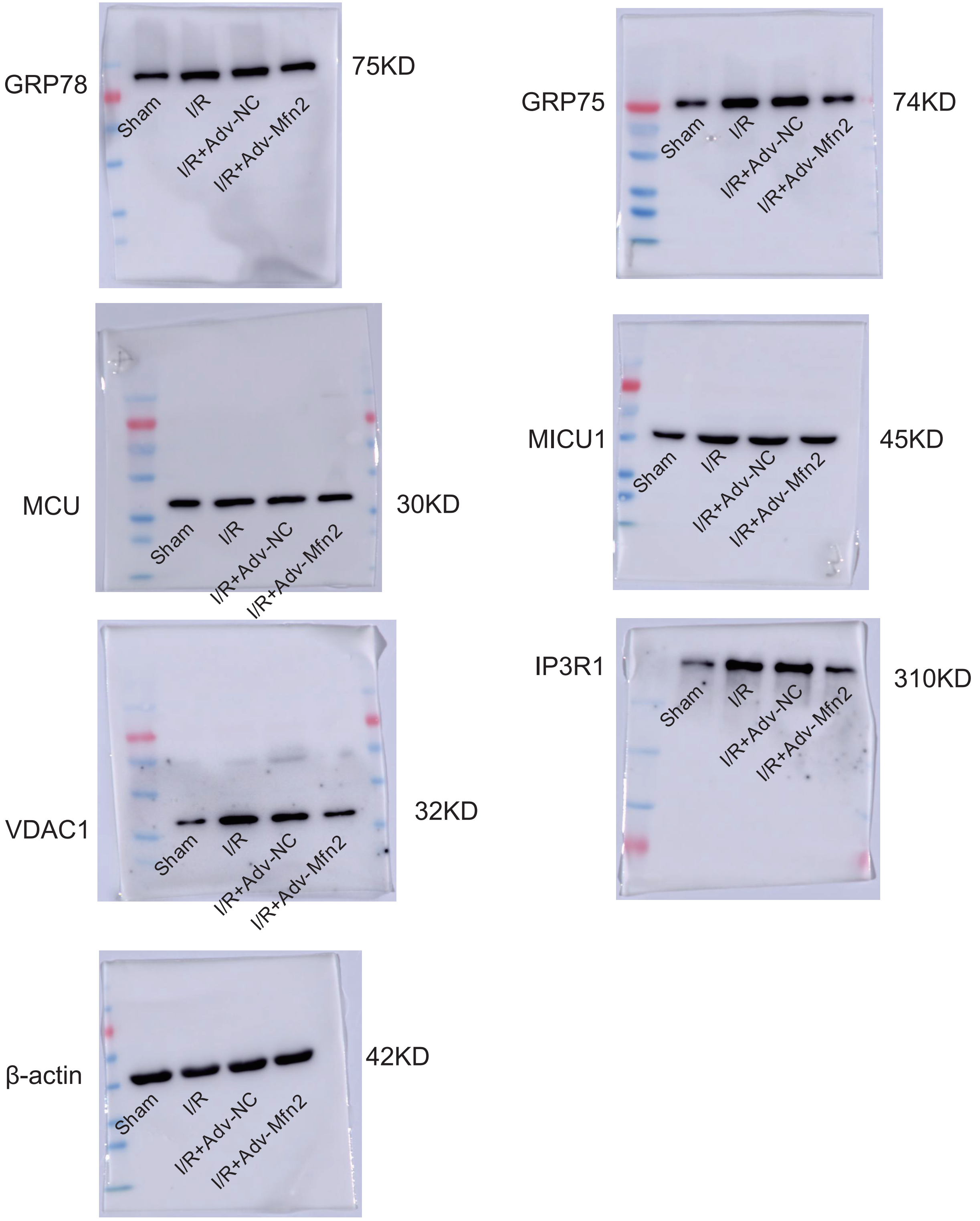

Figure S4. Images of expression of ER stress related protein detected by western blot at 24 h after renal I/R injury.
